# Characterizing approach behavior of *Drosophila melanogaster* in Buridan’s paradigm

**DOI:** 10.1371/journal.pone.0245990

**Published:** 2021-01-28

**Authors:** Rui Han, Tzu-Min Wei, Szu-Chiao Tseng, Chung-Chuan Lo

**Affiliations:** 1 Institute of Bioinformatics and Structural Biology, National Tsing Hua University, Hsinchu, Taiwan; 2 Institute of Systems Neuroscience, National Tsing Hua University, Hsinchu, Taiwan; 3 The Department of Life Sciences, National Tsing Hua University, Hsinchu, Taiwan; 4 Brain Research Center, National Tsing Hua University, Hsinchu, Taiwan; Biomedical Sciences Research Center Alexander Fleming, GREECE

## Abstract

The Buridan’s paradigm is a behavioral task designed for testing visuomotor responses or phototaxis in fruit fly *Drosophila melanogaster*. In the task, a wing-shortened fruit fly freely moves on a round platform surrounded by a 360° white screen with two vertical black stripes placed at 0° and 180°. A normal fly will tend to approach the stripes one at a time and move back and forth between them. A variety of tasks developed based on the Buridan’s paradigm were designed to test other cognitive functions such as visual spatial memory. Although the movement patterns and the behavioral preferences of the flies in the Buridan’s or similar tasks have been extensively studies a few decades ago, the protocol and experimental settings are markedly different from what are used today. We revisited the Buridan’s paradigm and systematically investigated the approach behavior of fruit flies under different stimulus settings. While early studies revealed an edge-fixation behavior for a wide stripe in the initial visuomotor responses, we did not discover such tendency in the Buridan’s paradigm when observing a longer-term behavior up to minutes, a memory-task relevant time scale. Instead, we observed robust negative photoaxis in which the flies approached the central part of the dark stripes of all sizes. In addition, we found that stripes of 20°-30° width yielded the best performance of approach. We further varied the luminance of the stripes and the background screen, and discovered that the performance depended on the luminance ratio between the stripes and the screen. Our study provided useful information for designing and optimizing the Buridan’s paradigm and other behavioral tasks that utilize the approach behavior.

## Introduction

The fruit fly *Drosophila melanogaster* serves as a good animal model for behavioral studies because of its complex behavior and abundant genetic tools [1]. Among many behavioral tasks designed for fruit flies, the Buridan’s paradigm and its variants have been extensively used to study various cognitive functions including visuomotor responses, phototaxis, and visual spatial memory [2–7].

In the Buridan’s paradigm, a single fly with shortened wings is placed into a round arena. Two black vertical stripes, serve as the visual cues, are attached on the wall of the arena and are separated by 180°. The fly is allowed to freely move in the arena. Without any training, a naïve fly tends to perform the approach behavior by walking back and forth between two black stripes persistently [4,8,9]. The movement patterns and behavioral preferences in the Buridan’s or similar tasks with different stimulus conditions were extensively investigated in 70’ and 80’ [5–7,10]. However, those studies were either focusing on the initial visuomotor responses at a time scale of seconds, or using different stimuli rather than two black stripes. In consequence, when we study the visual spatial memory of fruit flies at a time scale of minutes and use tasks that are developed based on the Buridan’s paradigm, the earlier results cannot provide sufficient metrics for comparison and optimization of the experimental setup.

In the present study, we systematically investigated the approach behavior with different stimulus settings at the time scale of minutes and revisited the question: what is the behavior preference of fruit flies in the Buridan’s paradigm? We tested the flies under three hypotheses for the behavioral preferences: (1) The flies prefer salient visual objects, (2) they are attracted by high-contrast edges, and (3) they like to approach dark regions. In the classical Buridan’s paradigm, which uses two narrow black stripes, all the three hypotheses lead to the same behavior preference, or the approach behavior toward the stripes. Therefore, the hypotheses are indistinguishable in this paradigm.

To address this issue, we modified the experimental setup by using different stripe widths so that we could dissociate the factors correlated with each of the hypotheses. We also introduced several behavior metrics that are more accurate in quantifying the detailed approach behavior than the metrics proposed earlier [9]. We further asked whether the luminance of the stripes or the luminance ratio between the stripes and the background screen constitute a major factor in the approach behavior. This was a previously un-explored experimental setup but critically affected the optimization of experimental apparatus.

## Materials and methods

### Fly strains

The fruit flies were incubated at 25°C with a 12:12 h light and dark cycle with a humidity level of ~50%. We used the wild-type strains, *w*^*+*^, which was obtained from Brain Research Center (BRC) in National Tsing Hua University (NTHU), Taiwan.

### The arena

**Fig 1:**
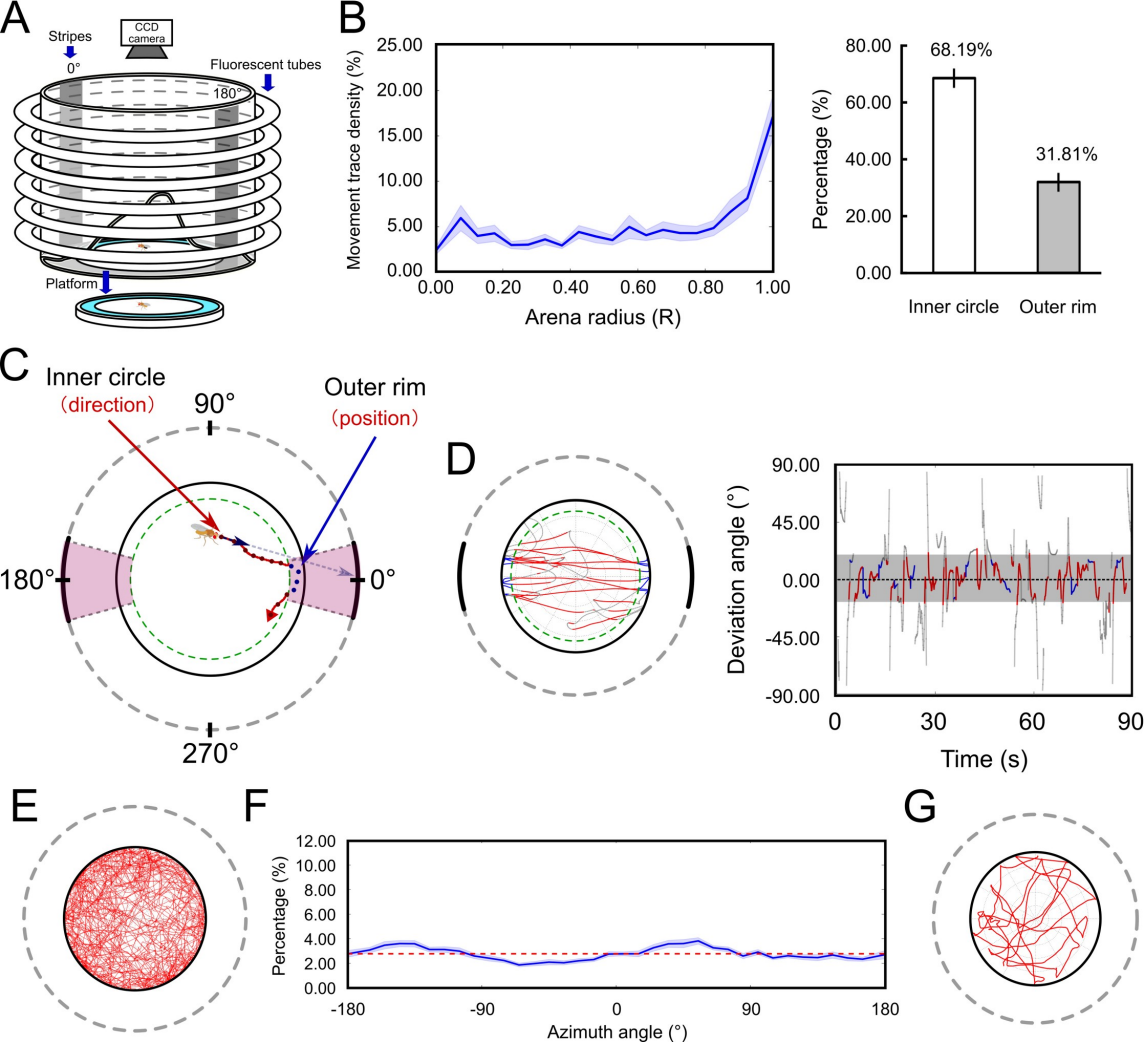
Schematics of the arena for the task and measurement of the deviation angle. (A) The behavioral arena. A circular platform was surrounded by water and by a screen illuminated by six circular fluorescent tubes. Two vertical dark stripes made of black cardboards were placed on the screen and served as the visual landmarks. In each trial, a single fruit fly was placed on the platform and allowed to move freely for 90 s. (B) (Left) The movement trace density (per unit area) for the *w*^*+*^ flies in the arena shown in (A). The x-axis is the normalized radial position *R* on the platform with 0.0 and 1.0 corresponding to the center and the edge of the platform, respectively. The shaded area indicates the standard error of the mean. The trace density is significantly larger in *R* > 0.85 than in *R* < 0.85. (Right) The percentages of time flies spent in *R* > 0.85 and *R* < 0.85. (C) In the behavioral task, we recorded the trace of the fruit fly and calculated the deviation angle for each video frame. In the inner circle (*R* < 0.85, the dashed green circuit), the deviation angle was determined by the projection of the movement vector on the screen (0° in this case), while in the outer rim (*R* > 0.85, between the dashed green circuit and the black solid circuit) the deviation angle was determined by the position of the fly in terms of the azimuth angle. See Materials & Methods for the detail. (D) Left: A typical trace of a fly with 30°-stripes. The red and blue lines indicate the stripe-fixation movement in the inner circle and the menotaxis in the outer rim, respectively. The gray lines indicate the non-approaching movement. Right: The movement trace represented by the deviation angle as a function of time. The colors follow the same definition as in the left panel. (E) Traces of 25 single flies in the all-bright (no stripe) condition for 90 s. (F) The distribution of azimuth angle of the same data as in the panel (E). The red line denotes the mean (≈ 2.78) of the distribution. The shaded area indicates the standard error of the mean. (G) A typical trace of a single fruit fly in the all-bright condition.

We established the behavioral tracking tool which followed the general design of previous researches [4]. The arena consisted of a round platform with a diameter of 100 mm and the platform was surrounded by water to prevent fly from escape (Fig 1A). The temperature of the arena was controlled at 25°C. The entire arena was surrounded by a screen made up of six white fluorescent tubes. The order product name of fluorescent tubes was MASTER TL5 Circular 55W/840 1CT/10 which emitted a cool white (CW) color. The power of each tube was 55.0 W. The entire screen was 190 mm in diameter and 180 mm in height. A CCD camera was mounted directly above the center of the platform and was used to record the movement traces of the flies using a Python 2.7 script developed in house. The frame rate of camera was 15 frames per second.

### Experiment procedure

Two or three days old wild-type flies (*Drosophila melanogaster*) were used in the present research and we shortened the wings one or two days before the experiments. The behavioral task was modified from the classic Buridan’s paradigm [3,4,10] (Fig 1A). In each trial, only one fly was placed on the platform and the fly was allowed to freely move for 90 s. In each trial, two vertical stripes, one centered at 0° and the other centered at 180°, were presented on the screen. The regions of the screen covered with the stripes are referred to as the “dark regions” or “dark stripes” while the rest un-covered regions are referred to as the “bright regions.” The stripes were made of thick black cardboards. The width of the stripes was fixed in each trial but different widths were used in the entire experiment. We used 11 different stripe widths: 0° (no stripes), 10°, 20°, 30°, 40°, 50°, 60°, 90°, 120°, 150° and 180° (stripes covering the entire screen). The widths 0° and 180° are referred to as all-bright and all-dark conditions, respectively.

To test the effect of luminance of the stripes and the background screen and luminance ratio (stripe luminance / background luminance) on the approach behavior, we replaced the cardboard by dark window films (made of polyethylene terephthalate, PET) which has a transparency of 5% in the visible and UV light ranges. The luminance of the stripes can be adjusted by stacking different layers of the films. We expect that stacking two layers of the films reduced the transparency to 5% × 5% = 2.5 × 10^−3^ and three layers to 2.5 × 10^−3^ × 5% = 1.25 × 10^−4^. This was verified by 10 repeated measurement of the luminance in each condition using a photometer. The average luminance of the background screen without window film was 1196 lux, and the average luminance after adding one, two and three layers of window films were 60 lux, 2.9 lux, and < 1 lux (below the measurement limit of the device), respectively. We also use the window films to adjust the luminance of the background screen, and the number of layers used for the background screen was always smaller than that used for the stripes, so that the stripes were always relatively darker than the screen. In the experiments, we tested 6 conditions of luminance as indicated by the layers of films for stripes vs. for screen: 3 vs. 0, 2 vs. 0, 1 vs. 0, 3 vs. 1, 2 vs. 1 and 3 vs. 2. The positions of the fly were recorded by a CCD camera at a speed of 15 frames per second for off-line analysis.

### Deviation angle and approach probability density

To quantify the approach behavior, we need to analyze the movement of each fly with respect to the stripes [11]. Intuitively, one would simply calculate the angle between the movement direction and the center of a stripe, or the deviation angle [2,9]. However, two issues need to be considered:

1. We discovered that the approach behavior consisted of two distinct movement patterns. When away from the edge of the platform, a fly tended to fixate on a stripe by moving toward it. After reaching the edge, the fly tended to spend a significant amount of time moving along the edge of the platform near the stripe (Fig 1B). This behavior can be described as menotaxis [12] as the fly kept the stripe in the center of the visual field of one eye. To take both movement patterns into account, we calculated the deviation angle in the outer rim (between 0.85 radius and the edge) for menotaxis, and in the inner circle (within 0.85 radius) for fixation separately (Fig 1C and 1D, S1 Fig). In the outer rim, the deviation angle was measured by fly’s position in terms of the azimuth angle with respect to the center of the closest stripe. In the inner circle, the deviation angle was calculated based on the movement direction as described below.
2. To calculate the deviation angle based on the movement direction, several different methods were implemented in various studies [2,9,13]. Most of these methods involving measurement of the angle between the vector representing the movement direction and the vector pointing to the center of a stripe. However, two issues may arise from these methods (S2 and S3 Figs). First, the maximum possible deviation angle varies and depends on the position of the fly. This issue causes a bias when plotting the distribution of deviation angle of a fly. Second, for a given width of the stripes, the range of deviation angle that indicates the approach behavior depends on the position of the fly. This issue complicates the way how approach behavior should be quantified. In the present study, we addressed these issues by modifying the definition of deviation angle. We first calculated the movement vector (the vector representing the displacement of the fly between two consecutive frames) and projected the vector onto the screen. Next, the deviation angle was defined by the azimuth angle between the point of the projection and the center of the closest stripe (Fig 1C).

For better presentation and analysis, after calculating the deviation angle in each video frame, we binned the data into 36 quantiles with each quantile spanning 10° in azimuth. Next, we calculated the percentages, *p*(*θ*), of the data falling in each quantile. *θ* denotes the azimuth angle of the center of each quantile, i.e. *θ* = 0°, 10°, 20°, …. 350°.

We determined the behavioral performance of the flies by calculating the approach probability density (*APD*) and approach strength (*AS*). *APD* measures the difference between approach toward the dark regions approach toward the bright regions. Specifically, the *APD* was calculated by subtracting the normalized *p*(*θ*)’s of the quantiles in the bright regions from the normalized *p*(*θ*)’s of the quantiles in the dark regions:

$$APD=\frac{\sum_{dark\;regions}p(\theta )}{number\;of\;dark\;quantiles\times 10}-\frac{\sum_{bright\;regions}p(\theta )}{number\;of\;bright\;quantiles\times 10}.$$ (1)

A positive *APD* value indicates a stronger approach (per degree) toward the dark regions than the approach toward the bright regions, while a negative value indicates the opposite. Zero indicates non-approaching movement that is toward the dark and bright regions with equal frequency. In addition, in the all-bright or all-dark conditions, the *APD* cannot be defined and therefore was not calculated.

### Radar plots and approach strength

To visualize the approach behavior, we created the radar plot, which indicated the frequencies of the movement toward each quantile. Due to the symmetric nature of the arena, we merged the data in the pair of quantiles that are 180° apart, e.g. quantiles centered at 90° and 270°, and calculated their averaged percentage. This procedure made the radar plot point-asymmetric. If a fruit fly did not exhibit any approach behavior, it would move toward all directions with equal probability, and the expected value of each quantile was 1 / 18 (~5.56%). A value that was significantly different from 1 / 18 indicated some form of approach behavior.

The radar plots allowed us to visually inspect the existence of approach behavior and its direction. We also defined a metric to quantify the approach behavior based on the radar plots. The radar plots usually form an ellipse and the longest axis, or the major axis indicates the direction of approach. The strength of approach can be measured by the ratio between the lengths of the major and the minor axes. To identify the major axis, one simply calculates the second moment of the ellipse with respect to any axis and the major axis is the one give rise to the smallest value. Based on this concept, we defined the approach angle (*σ*_*f*_) and the approach strength (*AS*). The approach angle (*σ*_*f*_) represents the direction of the strongest approach tendency and is defined by the angle *σ* of the axis that yields the smallest second moments *M*(σ) on the radar plot:

$$M(\sigma_{f})\equiv \underset{\sigma }{\min}\left(\sum_{i}{p(\theta_{i})}^{2}{sin}^{2}(\theta_{i}-\sigma )\right),$$ (2)

where *θ*_*i*_ is the angle of each quantile (0°, 10°, 20°, 30°, ….). The angle 0° is defined as the direction corresponds to the center of one of the dark stripes. *σ*_*f*_ can be obtained by taking the derivative of *M*(σ) with respect to σ.

$${\frac{dM(\sigma )}{d\sigma }|}_{\sigma =\sigma_{f}}=0.$$ (3)

Plugging *θ*_*i*_ and *p*(*θ*_*i*_) into this equation and solving for *σ* using trigonometric identities, we obtain

$$\sin\left(2\sigma_{f}+\phi \right)=0$$ (4)

and

$$\sigma_{f}=\frac{-\phi }{2}\;or\;\sigma_{f}=\frac{180{}^{\circ}-\phi }{2}$$ (5)

where *ϕ* is a variable depending on *p*(*θ*_*i*_) based on the following equations:

cosϕ=A/Kandsinϕ=B/K (6)

$$A=(\sum_{i=0}^{17}{\left(cos(20i{}^{\circ})\cdot p(10i{}^{\circ}\right)}^{2}))/2$$ (7)

$$B=-\sum_{i=0}^{17}{\left(sin(20i{}^{\circ})\cdot p(10i{}^{\circ}\right)}^{2})$$ (8)

$$K=\sqrt{A^{2}+B^{2}}.$$ (9)

The approach angle given in Eq (5) corresponds to two values. One gives rise to the minimum value of *M* (denoted as *M*_min_) and correlates with the length of the major axis. The other gives rise to the maximum value of *M* (denoted as *M*_max_) and correlates with the length of the minor axis. Finally, the approach strength (*AS*) is defined as follows:

$$AS=1-\frac{M_{min}}{M_{max}}.$$ (10)

The ratio between *M*_min_ and *M*_max_ indicates with the roundness of the ellipse, which correlates with the strength of approach. The approach strength *AS* is a value between 0 and 1. *AS* ≈ 1 for a long ellipse that is close to a line segment (perfect approach), and *AS* = 0 for an extremely round ellipse that is close to a circle (no approach). To determine the minimum level of *AS* that represents a statistically significant approach behavior, we performed simulations by generating random movement traces for 25 simulated flies and calculated their approach strength. The mean approach strength was 0.224 and the standard deviation was 0.160 for the simulated data. We took two standard deviations above the mean (*AS* = 0.544) as our statistical criterion for strong approach behavior and one standard deviations above the mean (*AS* = 0.384) for weak approach behavior. No significant approach behavior is considered when *AS* is smaller than 0.384.

We stress that *APD* and *AS* serve different purposes in quantifying the approach behavior. *APD* measures the tendency of approach toward the dark versus the bright regions. The value is normalized by the width of each region. By contrast, *AS* measures the tendency of approach toward any part of the screen and is independent of the presence of the dark and bright regions. The exact azimuth angle of the approach is specified by *σ*_*f*_.

### Analysis of locomotion

To quantify whether different stripe widths led to different levels of locomotion, we analyzed the average movement speed and activity level of the fruit flies (Table 1). The average speed was calculated by dividing the total movement distance by the duration of a trial. The activity level was defined as the percentage of time frames that a fruit fly moved in a trial.

**Table 1:** Activity levels and movement speeds of the w+ flies for different widths of the dark stripes, different layers of window films, for the 30° dark stripes.

Dark stripe width	Activity level (%)	Speed (mm/s)
0° (all bright)	64.80 ± 4.11	9.09 ± 0.70
10°	46.00 ± 3.12	7.72 ± 0.51
20°	59.84 ± 3.42	10.66 ± 0.52
30°	61.49 ± 3.23	9.92 ± 0.60
40°	53.97 ± 4.43	7.63 ± 0.63
50°	56.79 ± 3.95	7.27 ± 0.64
60°	58.27 ± 3.87	8.55 ± 0.55
90°	61.58 ± 4.24	8.11 ± 0.68
120°	67.07 ± 3.51	8.78 ± 0.61
150°	61.60 ± 3.58	8.63 ± 0.65
180° (all dark)	60.52 ± 4.11	10.71 ± 0.86
30° (3-layer stripe)	56.12 ± 3.51	9.54 ± 0.60
30° (2-layer stripe)	75.87 ± 4.19	12.79 ± 0.41
30° (1-layer stripe)	71.19 ± 3.93	11.65 ± 0.73
30° (3-layer stripe + 1-layer screen)	62.56 ± 4.51	9.27 ± 0.53
30° (2-layer stripe + 1-layer screen)	82.22 ± 3.21	13.64 ± 0.34
30° (3-layer stripe + 2-layer screen)	73.79 ± 3.40	11.56 ± 0.47
30° (one dark stripe)	61.92 ± 3.47	9.00 ± 0.44
30° (one bright stripe)	48.91 ± 4.17	6.50 ± 0.59

### Statistical analysis

A total of 576 flies were used in the study but we removed 21 individuals which had significantly less locomotion than the others. The removal criterium was two standard deviations below the group mean of the activity level or the speed. Therefore, 555 flies were included in the analysis with 20–25 flies for each test condition.

The statistical analyses were performed using Statistical Product and Service Solutions 22.0 (SPSS 22.0). A multi-factor within group analysis of variance was used for analyzing the probability density, and a mix variance analysis was used for evaluating differences between groups of different experimental conditions.

### Effects of gender and recovery period

The present study used both genders and 1–2 days of recovery period (the time between wing-shortening and behavioral experiment). To evaluate the effect of gender and recovery period on the behavior performance, we conducted a small set of experiment (30° stripes, 25 trials for each condition) separately for the male and female flies, and also for 1, 3 and 5 days of recovery period (S4 Fig). No significant difference in *APD*, activity level and speed were observed between female and male flies. For different recovery periods, there were neither differences in *APD* nor activity level. However, flies with 5-day recovery period exhibited slight but statistically significant reduction in the speed comparing to the flies with 1-day recovery period.

## Results

### The arena and trace distribution

We constructed the behavioral arena (Fig 1A) based on the previous studies [4] (see Materials and Methods). We first examined the behavior of the flies in the arena without showing any stripe and confirmed that the flies moved randomly on the platform without significant direction preference. They spent more time moving along the edge as evident by the denser distribution of traces in the outer rim area than in the inner circle. This observation is consistent with the common notion of the open-region avoidance behavior of fruit flies, in particular for those without functioning wings [9,14]. This result indicated that the arena did provide a homogeneous illuminating environment without any cue that can elicit directional bias (Fig 1E–1G).

### Three hypotheses for the approach behavior

**Fig 2:**
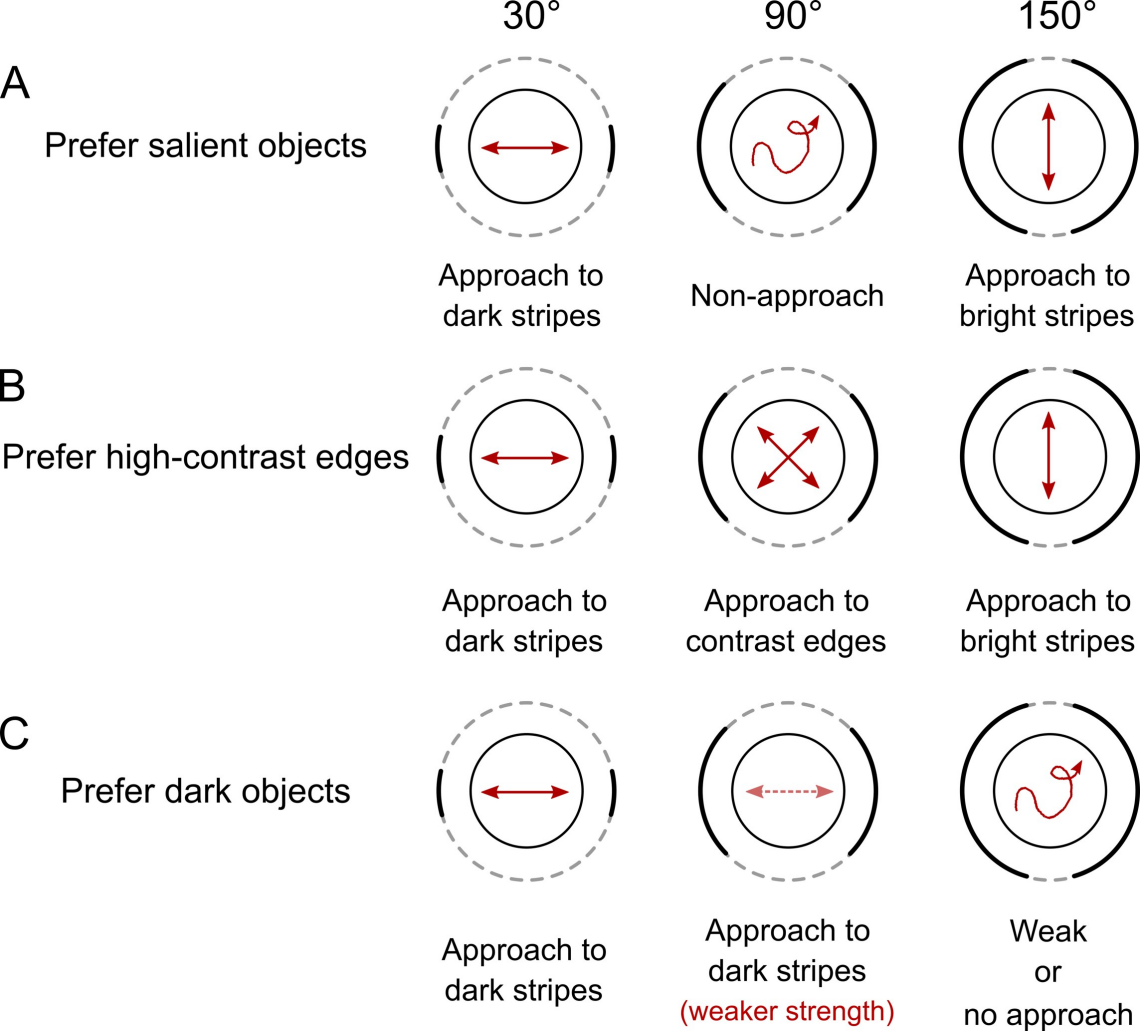
Three hypotheses for the approach behavior. (A) The saliency hypothesis. If the wing-shortened flies prefer salient objects, the movement would approach visually salient objects (left: dark stripes, right: bright stripes). The flies would not perform approach behavior when the dark and bright stripes are equally large (middle). (B) The contrast hypothesis. If flies are attracted by high-contrast stimuli, they would approach either narrow dark stripes (left) or narrow bright stripes (right) just as in the saliency hypothesis. However, when the dark and bright stripes are equally large (middle), the flies would approach the four edges. (C) The shade (negative phototaxis) hypothesis. If the flies prefer dark regions, they would always approach the dark stripes no matter their size (left and middle). However, when the most of the screen is covered by the dark stripes (right), the approach behavior would become weak or even disappear. All three hypotheses predict the same approach behavior in the classical Buridan’s paradigm (right) and the hypotheses can only be tested and verified when the stripes of different sizes are used.

The approach behavior of fruit flies in the Buridan’s paradigm is known to be robust. However, the underlying behavioral mechanism was still unclear and not systematically tested. We proposed three hypotheses for the mechanism: (1) The saliency hypothesis. In this hypothesis, the flies tend to move toward any visual pattern that stands out of the background, whether it be a dark stripe in a bright background, or a bright stripe in a dark background (Fig 2A). (2) The contrast hypothesis. In this hypothesis, the flies tend to move toward the high contrast regions, or the edges of stripes. Therefore, when the flies are attracted by the edges of a narrow stripe, they appear to move toward the stripe (Fig 2B). (3) The shade, or the negative phototaxis hypothesis. In this hypothesis, the flies like to approach dark regions, regardless of their size, saliency or contrast (Fig 2C). In the classic Buridan’s paradigm in which two narrow dark stripes are placed on the screen, all three hypotheses yield the same approach behavior. In order to separate the factors that are associated with each hypothesis, we tested the flies with various widths of the stripes. For example, if the stripes are widened from the original 30° to 90°, the screen is divided to four regions (two dark, two bright). In this case the saliency hypothesis would predict no specific approach behavior because there is no obvious foreground object. By contrast, the other two hypotheses predict specific but different approach behaviors. The contrast hypothesis predicts a movement tendency toward the four edges between the dark and bright regions while the shade hypothesis predicts that the movements are only toward the dark regions and the distribution of movement traces is broader than that in the condition of 30° dark stripes (Fig 2, left and middle columns). Finally, if we increase the width of the dark stripes to 150°, which effectively makes the rest of bright regions salient objects, the three hypotheses predict different behavioral outcomes again. The saliency hypothesis predicts a clear approach behavior on the 30° bright stripes. The contrast predicts the same as the saliency one does. However, the shade hypothesis predicts no or very weak approach behavior because most region is dark (Fig 2, right column).

### Approach behavior in example trials

**Fig 3:**
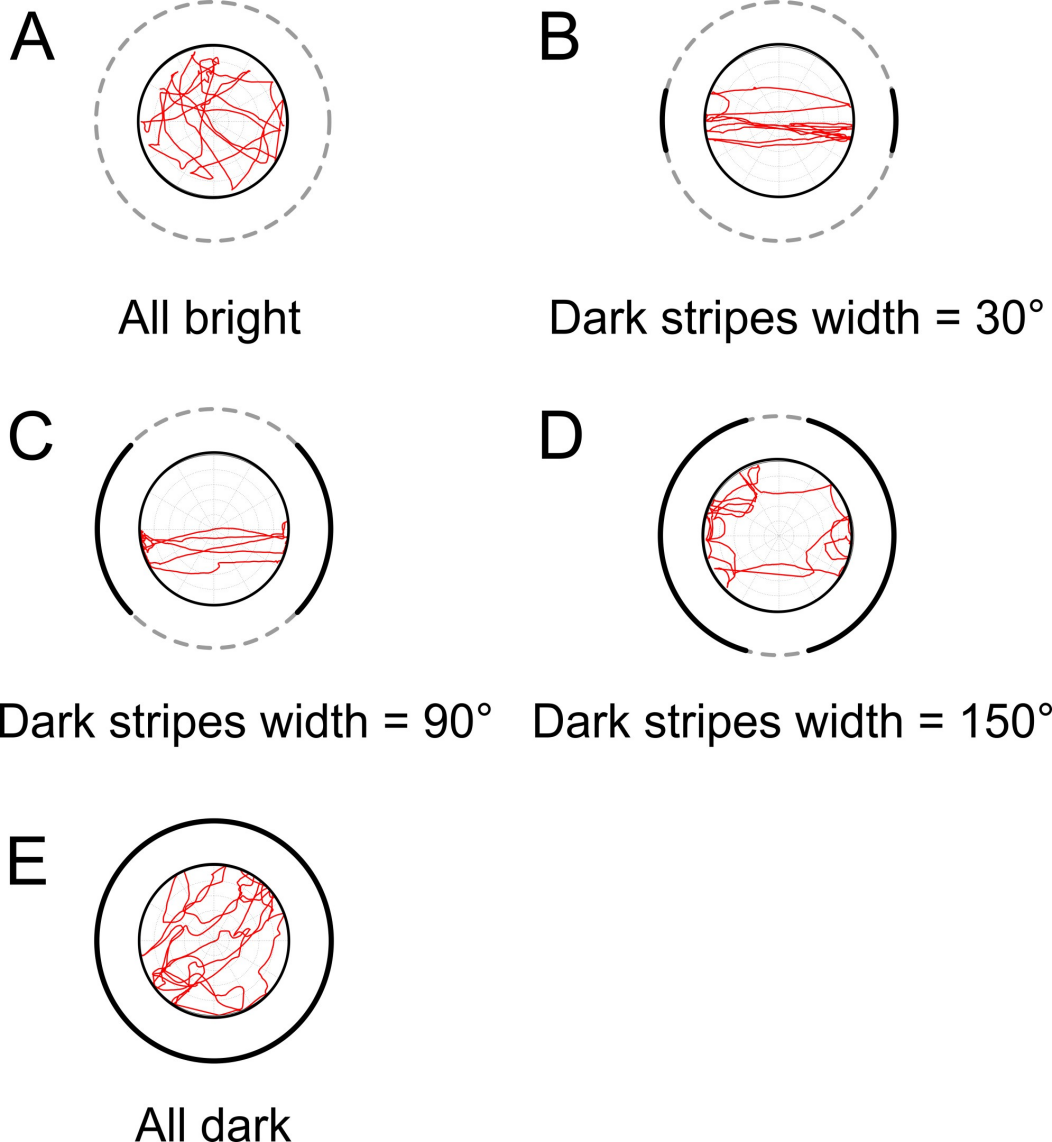
Example movement traces of the *w*^*+*^ wild-type flies in five different stimulus conditions. (A) In the all-bright condition, in which no dark stripe was present, the fly did not exhibit any approach behavior. (B)-(D) Example movement traces for different dark stripe widths (30° (B), 90° (C) and 150° (D)). The approach behavior presented in all conditions, include those with very wide stripes. (E) No apparent approach behavior was observed for the all-dark condition.

To test the three hypotheses, we first examined the behavior of the fruit flies (*w*^*+*^) in different conditions of stripe widths in several example trials (S1 Video). Next, we calculated the approach probability density (*APD*) (see Materials and Methods) for individual flies (Fig 3; S5 Fig). The *APD* was large and positive for narrow dark stripes (20°-40°), but reduced when the widths of the stripes increased. Although the *APD* was low for wide dark stripes, visual inspection still revealed clear approach patterns that were toward the dark stripes (Fig 3C and 3D). No approach of the stripe edges was observed. The behavioral preference of the example trials was consistent with the shade hypothesis (Fig 2C).

### The approach behavior at the population level

**Fig 4:**
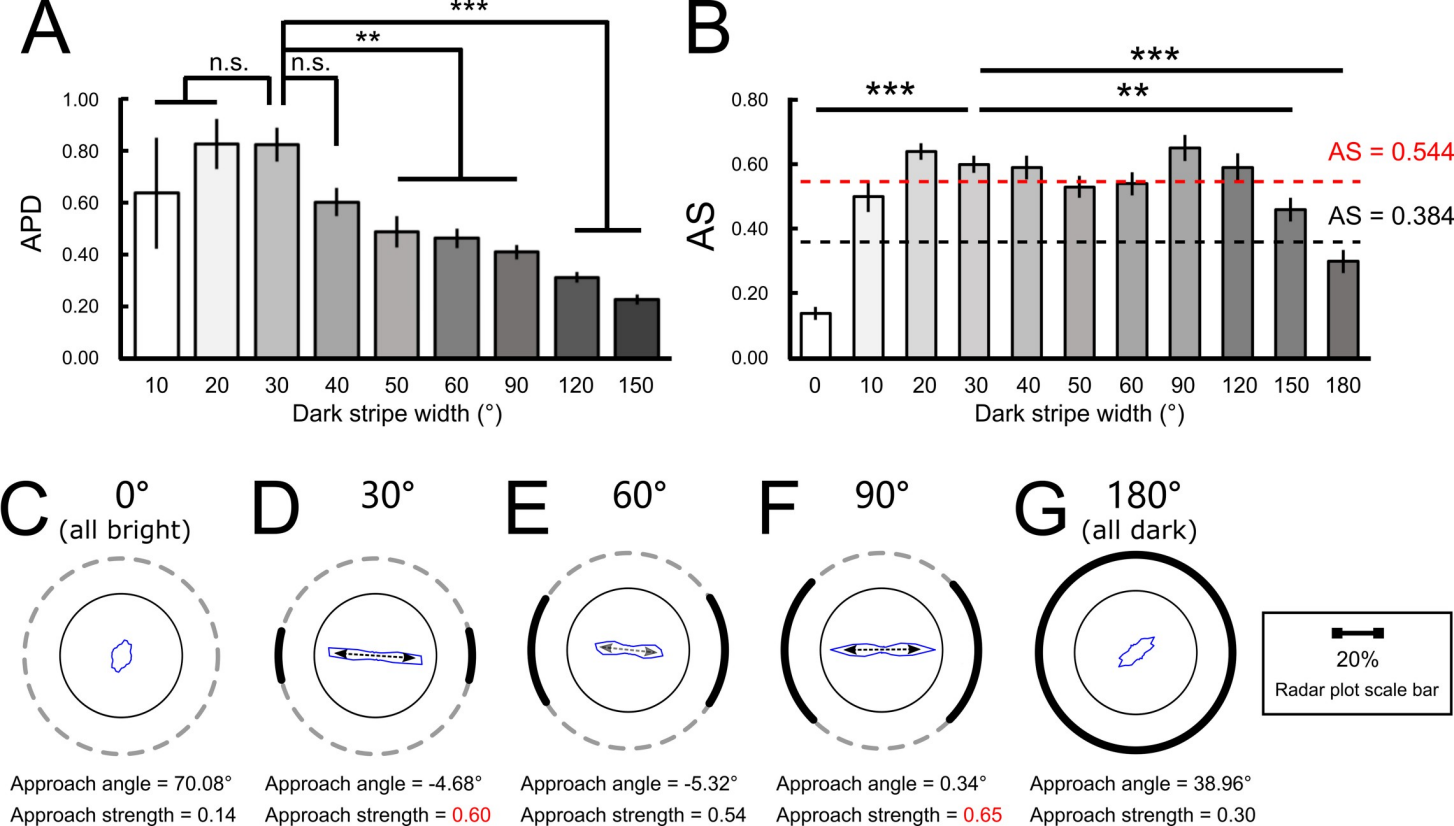
The population-averaged approach behavior for different stripe widths. (A) The mean population *APD* (approach probability density) indicates the difference between approach tendency on the dark region and the bright region, normalized by the angular span of each region. Positive *APD* indicates the tendency of dark stripe approach. While the stripe width of 20° or 30° gave rise to the largest *APD*, significant approach behavior (*APD* > 0) can still be observed for all stripe widths. (B) The mean *AS* (approach strength), which indicates the strongest approach toward any direction regardless of the position of the stripes. *AS* that falls below the black dashed line is considered as no significant approach. *AS* that falls between the black and red dashed line is considered as significant but weak approach. *AS* that is above the red dashed line indicates strong approach. Again, all stripe widths gave rise to significant approach behavior. (C)-(F) The radar plots for the all-bright condition and three representative stripe widths (30°, 60° and 120°). The approach was all toward the center of the stripes regardless of the width of the dark stripe, as evident by the small approach angles.

Next, we analyzed the population *APD* and *AS* (Fig 4A and 4B). We found that although the activity level and speed did not vary significantly across most stripe widths (see Table 1), the *APD* changed significantly. The *APD* was largest when the stripes width was 20° or 30° and was smaller for narrower or wider stripes (Fig 4A). But the positive *APD* values indicated that the flies approached the dark stripes for all stripe widths. Further analysis showed that movements in the outer rim and in the inner circle both contribute to *APD* comparably (S6 Fig).

One should note that *APD* is a normalized measure which takes the widths of the dark and bright regions into account. Specifically, *APD* indicates the “density” of approach per degree of the dark stripes, but not the total approach strength. So, a wide stripe may potentially “dilute” the *APD*. Therefore, we examined *AS* (Fig 4B), which is independent of the widths of the stripes and is better for indicating the total approach strength. We discovered that, except for all-bright and all-dark conditions, *AS* was significant for all widths of the stripes. Interestingly, wide stripes (60°-120°) produced *AS* with magnitudes comparable to those by the narrow stripes (20°-40°). To further examine the movement patterns of flies in more detail, we plotted the radar plots, which indicate the frequency of movement toward all angles in the 36 quantiles (Fig 4C–4F; S7 Fig). We discovered that the approach was indeed very concentrated on the center of the stripes regardless of their widths, as evident by the small approach angles. Again, the result did not support the saliency and contrast hypotheses. Note that for the condition of 90° dark stripes, the approach behavior was still strong (approach strength = 0.65) and the major axis of the plot points toward 0°, rather than toward ±45° as the contrast hypothesis may predict.

### Distributions of the deviation angle

**Fig 5:**
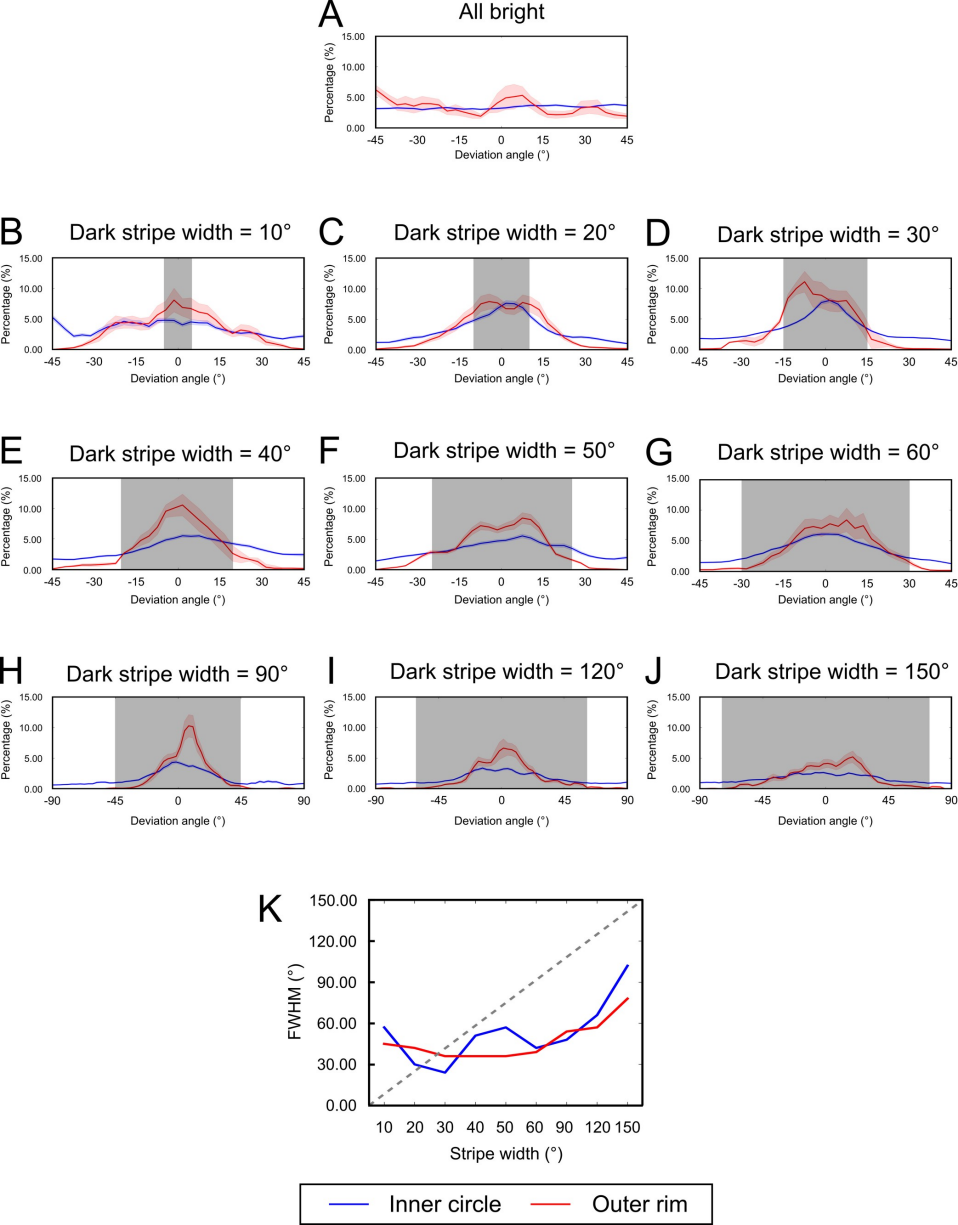
Detailed approach patterns as revealed by the distributions of deviation angle. (A)-(J) The distribution of deviation angle for all stripe widths. The shaded area represents the span of dark stripes. The distributions are plotted for two circular regions on the platform. Blue: the central circle (within 0.85 radius). Red: outer rim (outside 0.85 radius). Each curve is normalized by the time flies spent in the corresponding region. (K) The full width at half maximum (*FWHM*) of the distributions of deviation angle shown in (A)-(J) as a function of the stripe width. The curves suggest that the approach was highly concentrated around the centers of the stripes in a range about 30°-60° regardless of the width of the dark stripes except for the 150°. The curves would overlap the dashed line *(FWHM* = stripe width) if the flies approached the entire span of the dark stripes.

*APD* and *AS* provide concise information about the approach behavior. However, it would be more informative if we can examine the detailed distribution of the deviation angle around the dark stripes. To this end, we plotted the distributions of the deviation angles separately for the two regions (central circle, outer rim) (Fig 5A–5J). We also measured the full width at half maximum (*FWHM*) of each distribution and plotted *FWHM* as a function of the widths of the dark stripes (Fig 5K).

For all stripe widths, the distributions always peaked at the center of the dark stripe. However, we discovered a couple interesting trends regarding the widths of the distributions. First, a wider stripe helped to reduce the width of the distribution. When the dark stripes were only 10° or 20° wide, the flies could not focus their approach in the azimuth range of the dark stripes and the distributions extended into the surrounding bright regions considerably (Fig 5B and 5C). However, when the stripe width further increased, the distribution became narrower and was mostly covered by the dark stripes (Fig 5D and 5E). As the width of the stripes further increased, the distribution only widened slightly, and only covered the central portion of the stripes (Fig 5F–5J). This observation suggested that the approach behavior was subtler than that predicted by the simple shaded theory. If the flies simply preferred moving toward the dark objects, the widths of their deviation angle distributions should also be in proportion to the widths of the dark stripes. However, this was not the case (Fig 5K). We discuss the implication of this observation in the Discussion section. If we simply compare *FWHM*, stripes of 20° to 30° width induced the most concentrated approach behavior.

Fig 5 does not show the relative frequency between the inner circle and outer rim as each curve was normalized individually. To inspect the relative frequency, we normalized both curves by the time flies spent in the entire arena (S8 Fig). We found that the outer rim contributed slightly more to the approach behavior than the inner circle does. But the general conclusion stated above does not change.

### The role of luminance of the stripes in the approach behavior

**Fig 6:**
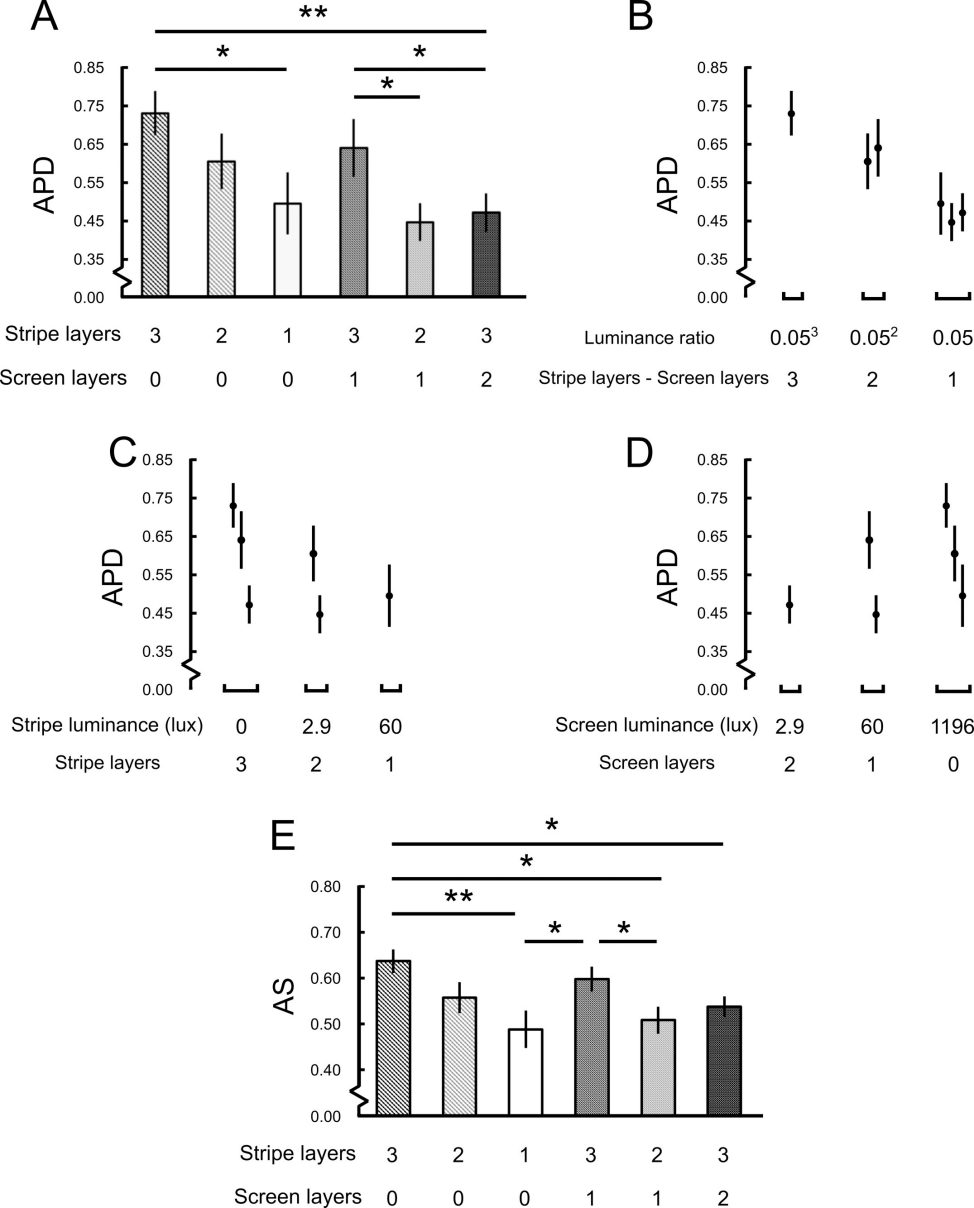
Influence of the luminance of the stripes and the background screen on the approach behavior. (A) The mean population *APD* for different luminance of the stripes and screen. The luminance was manipulated by the number of dark window films (5% transparency in visible lights and UV) used for the stripes or the screen, as indicated in the abscissa. A larger number of layers represents a darker stripe or screen. (B) The *APD* as a function of the luminance ratio of the stripes and the screen, as indicated by the difference in the number of films used. A strong positive correlation can be observed. (C) and (D) The *APD* as a function of the luminance of the stripe and the screen, respectively. (E) The *AS* for different luminance of the stripes and screen. A similar trend as in A can be observed.

We further investigated how other physical properties such as the luminance of the stripe may affect the behavior. Here we tested two hypotheses: 1) the approach behavior depends on the luminance of the stripes, and 2) the approach behavior depends on the luminance ratio between the stripes and the background screen. To this end, we vary the luminance of the stripes and the background screen by using different layers of dark window films (Fig 6A; S2 Video). By plotting the *APD* against the luminance ratio and the luminance, we concluded that the approach behavior correlated with the luminance ratio between the stripes and the screen (Fig 6B–6D). *AS* also exhibited the similar trend with that of *APD* (Fig 6E).

## Discussion

In the present study, we sought to provide a behavioral-level explanation of the visual approach in the fruit flies. We tested how widths and the luminance of the stripes affected the approach behavior. We discovered that among the different explanations of the approach behavior, our result supported the shade hypothesis, in which the flies prefer to approach dark objects. We introduced new metrics that worked better than previous metrics for evaluating the approach behavior of wide stripes. We found that the stripes with a width of 20°-30° were most effective in eliciting approach behavior, and the flies approached the entire azimuth range of the dark stripes. Wider stripes also elicited the approach behavior with comparable strengths, but the flies only approached the central part of a stripe with relatively weak approach toward the peripheral part. Further tests discovered that the approach is strongly correlated with the luminance ratio of the stripes and the background screen, but not the luminance of either the stripes or the screen.

The approach toward the center of a wide stripe is a surprising observation and is worth discussion. Although the behavioral results of the present study support the shade hypothesis, it would predict that the approach is toward the entire azimuth range of a wide stripe. Therefore, the observation of approaching toward the central part of a wide stripe suggested that other behavioral drives might be involved. Could the bright regions (which were narrower than the stripes when they were larger than 90°) produce certain “repulsive” effect that drove the flies toward the central portion of the dark stripes? Do the specific compound eye structure or the visual system properties play roles here? Novel experimental settings need to be developed in order to study this phenomenon.

Some early studies [5,7] with different experimental setting also observed the preference of flies in moving toward a black stripe on a white background. Moreover, the study discovered an edge-approaching effect when the stripe was very wide or very narrow. The effect was also dependent on the radial position of the flies. Furthermore, in Wehner 1981 [7], the author tested the approach behavior of fruit flies in an arena with one 180° wide black stripe. The results showed that the edge-approaching behavior occurred when the height was more than 10°. We did observe such an edge-approaching behavior in our arena with a single wide dark stripe, a setting similar to Wehner 1981 [7]. However, we did not find such behavior in our Buridan’s paradigm (two stripes) for the stripes of any size, nor did we find such behavior for different radial positions of the flies. The discrepancy might arise from the experimental design. For example, in Wehner 1972 [5] and Horn & Wehner 1975 [6], each fly started the walk from the center of the platform and was removed from the platform once the fly reached the edge. Therefore, they tested the “initial” visuomotor response of flies in some sense. In the present study, the flies were allowed to move on the platform continuously for 90 s. This way we observed a longer-term behavior of the flies than Wehner 1972 [5] and Horn & Wehner 1975 [6] did. Further study is required in order to identify the actual factors that lead to the discrepancy.

One more interesting issue to discuss is the early studies of fruit fly choice experiments by Götz [10,15], in which the author demonstrated strong and persistent approach behavior for salient objects. The author argued that the guided orientation task with only one landmark tended to elicit stronger fixation behavior than the Buridan’s task did because flies in the latter task often stopped approaching one landmark, turned their bodies, and approached another landmark. Indeed, our test showed that if we presented only one dark stripe in our arena, the flies exhibited stronger approach performance than in the two-stripe Buridan’s setting (S9A–S9D Fig; S1 and S3 Videos). The result is consistent with Götz’s. However, if we presented one bright stripe, the flies did not approach at the bright stripe at all (S9A and S9B and S9E Fig; S3 Video). Same for two bright stripes as shown in Figs 4 and 5. Therefore, when we presented with bright stripes or dark ones, no matter whether there were one or two stripes, flies always prefer the latter.

In the present study, we only consider the explanation of approach at the behavioral level. The neural mechanism underlying such shade-seeking behavior, or negative phototaxis, is still not clear. In a previous study, photophobism has been observed in larva [16], but the wild-type adults showed positive phototaxis. Interestingly, another study that used the wingless adult flies showed negative phototactic behavior [17]. Therefore, we suggest that the shade-seeking behavior may be specific to wing-shortened fruit flies and the loss of the ability to fly may play a major role in such a tendency. One possible explanation for the shade-seeking behavior is that the dark stripe resembles an escape route or appears to be a safe place to hide. Moreover, several recent studies on spatial orientation memory of fruit flies [2,3,18,19] have utilize the approach or fixation behavior as the behavioral measure, and the central complex [20] has been found to be strongly associated with the spatial orientation memory. Therefore, the present behavior level study may provide detailed metrics that helps with extending the computational models of the central complex [14,19,21–26] and linking the neural circuit level mechanisms with the behavior.

It is interesting to note that, fruit flies with different genetic backgrounds may perform differently in Buridan’s paradigm. For example, although the white-eyed *w*^*1118*^ flies are known to have poor vision, in another ongoing study we discovered that this types of flies actually perform decently in an arena that used dimmer green LEDs as the light source [14]. Therefore, the optimal experiment setup may well depend on the strain of the flies and is worth a systematic study in the future.

## Supporting information

S1 Fig Comparison between flies which exhibited higher density of movement trace in the inner circle than the outer rim (A)-(C) and flies which were opposite (D)-(F). (A) This subgroup of flies spent majority of the time in the inner circle. (B) *APD* based on two different methods for calculating the deviation angle. In Method 1, the deviation angle was calculated based on the movement vector regardless of the location on the platform. In Method 2, which was used in the present study, the deviation angle was calculated differently in the inner circle (< 0.85 radius) and in the outer rim (> 0.85 radius). See Materials & Methods for detail. There is no significant difference in *APD* between the two methods. (C) Movement traces from five representative flies in this subgroup. (D)-(F) same as in (A)-(C) but for the subgroup of flies which spent majority of the time in the outer rim. Method 2 gave rise to a large *APD* but not Method 1, despite the visually significant approach patterns as seen in five representative traces shown in (F).
(TIF)

S2 Fig Comparison between our definition of deviation angle and the definition used in (Columb et al 2012). (A) Using Columb’s method, the maximum possible deviation angle α depends on the position of the fly. α < 90° at the position shown here. (B) Using Columb’s method, depending on the position of the fly, the same deviation angle may or may not indicate approach toward the stripes. (C) The distribution of deviation angle in the all-bright condition, in which the flies performed non-approaching movement without any directional preference. Our method (blue) gave rise to a flat curve that covers all angles (0°-90°), while Colomb’s method gave rise to a curve with a dip at the large angle region due to the issue illustrated in (A). (D) The distribution of deviation angle in the 30°-stripes condition. In our method, angles that are below 15° (shaded area) indicate the approach behavior. However, in Colomb’s method, due to the issue illustrated in (B), it is difficult to define an angular value as the criterium for the approach behavior. (E) When a fly approaches a location on the screen and moves across the platform, our method produces a constant deviation angle indicating the azimuth angle of the approached position, while Colomb’s method produces a curve with a large change in angle.
(TIF)

S3 Fig Comparison between our behavioral metrics and the metrics defined in Colomb et al. 2012. (A) In our method, we first calculate the deviation angles for each video frame and then derive *APD* and *AS* from the deviation angles. (B) In Colomb’s method, in addition to calculating the median deviation angle, another metric called “number of walks” was also used. This metric measures the number of walks a fruit fly made between the shaded areas. However, when the stripes become very wide, some walks that still clearly approach the dark stripes may out count in (C) Due to the issue illustrated in (B), the number of walks declines as the width of stripes increases. (D) Our *AS* still gave rise to large values even for wide stripes.
(TIF)

S4 Fig Genders and the recovery period do not affect the motor functions and the approach behavior. (A) Comparison between female and male for *APD*, activity level and speed. (B) Comparison between different recovery periods (1, 2 and 3 days).
(TIF)

S5 Fig Example traces and the *APD* for all tested stripe widths. (A) Without any stripe (all-bright condition), the fly did not exhibit any approach behavior. (B)-(J) Example traces and the time-resolved *APD* for different dark stripe widths. (K) The fly did not exhibit any approach behavior in the all-dark condition.
(TIF)

S6 Fig The population mean *APD* and the time–resolved *APD* for all tested stripe widths. (A) The *APD* separately calculated for the inner circle and the outer rim. Both regions contribute to the *APD* significantly. (B)-(J) Time-resolved *APD* for all tested stripe widths. *APD* is not defined for the all-bring or all-dark conditions. The shaded area indicates the standard error of the mean.
(TIF)

S7 Fig The population-averaged radar plots for all conditions. Dark stripes of all sizes elicited significant approach strength with the approach direction toward the center of the dark stripes.
(TIF)

S8 Fig Detailed approach patterns as revealed by the distributions of deviation angle. Similar to Fig 5, but both curves in each plot are normalized by the total time spent in the entire arena. Ratio between the time flies spent in the outer rim and inner circle for each condition is labeled under each corresponding plot.
(TIF)

S9 Fig Approach behavior for one single dark stripe or bright stripe. (A) Example movement traces for one dark stripe, two dark stripes (Buridan’s paradigm) and one bright stripe. (B) The population mean *APD* for all three conditions. (C)-(D) Distributions of deviation angle for all three test conditions.
(TIF)

S1 Video Dark stripe width = 0°, 10°, 20°, 30°, 40°, 50°, 60°, 90°, 120°, 150°, 180°, each condition lasts 90 s. The dark stripes existed in the upper and bottom of the screen.
(MOV)

S2 Video Dark stripe width = 30°, material = window films, 3-layer stripe & no layer screen, 2-layer stripe & no layer screen, 1-layer stripe & no layer screen, 3-layer stripe & 1-layer screen, 2-layer stripe & 1-layer screen, 3-layer stripe & 2-layer screen, each condition lasts 90 s. The dark stripes existed in the upper and bottom of the screen.
(MOV)

S3 Video Stripe width = 30°. One dark stripe and one bright stripe. The dark or bright stripe existed in the upper of the screen.
(MOV)
