# Supplementary figures and images for: Characterizing approach behavior of Drosophila melanogaster in Buridan’s paradigm
Source: PLoS One. 2021 Jan 28;16(1):e0245990. doi: 10.1371/journal.pone.0245990 (PMC7843020; doi:10.1371/journal.pone.0245990)

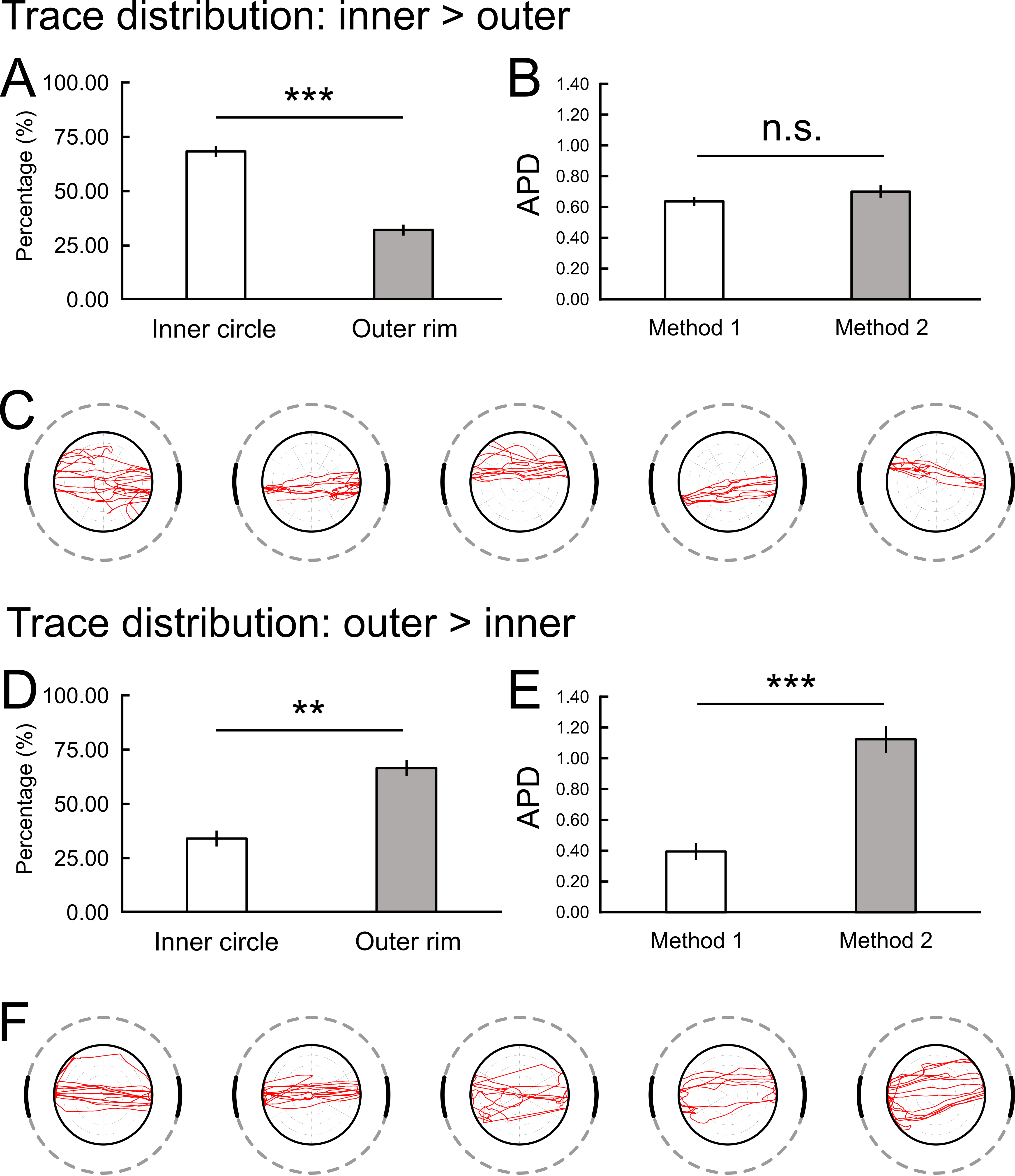

Supplement: S1 Fig — Comparison between flies which exhibited higher density of movement trace in the inner circle than the outer rim (A)-(C) and flies which were opposite (D)-(F). (A) This subgroup of flies spent majority of the time in the inner circle. (B) APD based on two different methods for calculating the deviation angle. In Method 1, the deviation angle was calculated based on the movement vector regardless of the location on the platform. In Method 2, which was used in the present study, the deviation angle was calculated differently in the inner circle (< 0.85 radius) and in the outer rim (> 0.85 radius). See Materials & Methods for detail. There is no significant difference in APD between the two methods. (C) Movement traces from five representative flies in this subgroup. (D)-(F) same as in (A)-(C) but for the subgroup of flies which spent majority of the time in the outer rim. Method 2 gave rise to a large APD but not Method 1, despite the visually significant approach patterns as seen in five representative traces shown in (F). (TIF) [file pone.0245990.s001.tif]

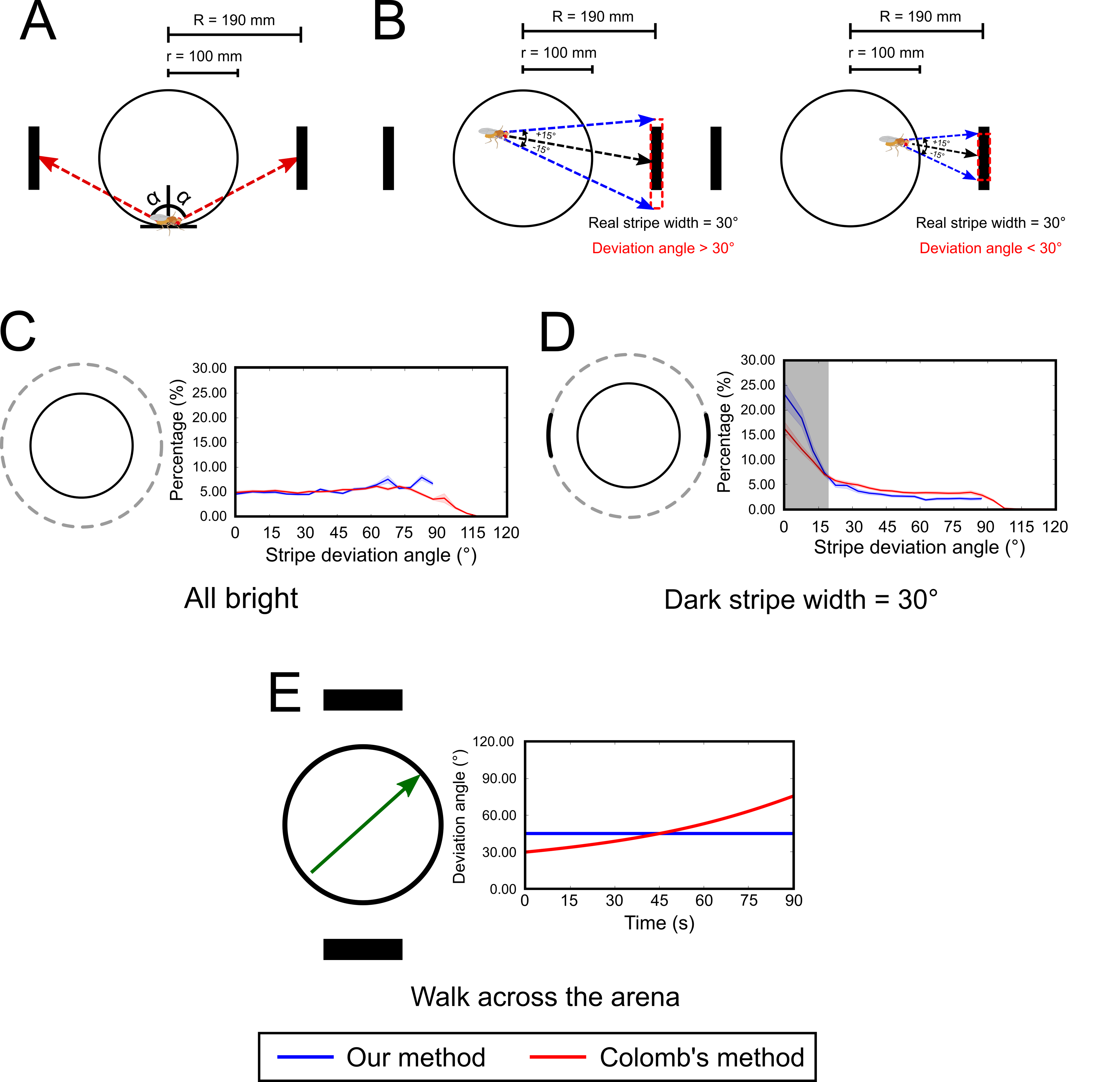

Supplement: S2 Fig — (A) Using Columb’s method, the maximum possible deviation angle α depends on the position of the fly. α < 90° at the position shown here. (B) Using Columb’s method, depending on the position of the fly, the same deviation angle may or may not indicate approach toward the stripes. (C) The distribution of deviation angle in the all-bright condition, in which the flies performed non-approaching movement without any directional preference. Our method (blue) gave rise to a flat curve that covers all angles (0°-90°), while Colomb’s method gave rise to a curve with a dip at the large angle region due to the issue illustrated in (A). (D) The distribution of deviation angle in the 30°-stripes condition. In our method, angles that are below 15° (shaded area) indicate the approach behavior. However, in Colomb’s method, due to the issue illustrated in (B), it is difficult to define an angular value as the criterium for the approach behavior. (E) When a fly approaches a location on the screen and moves across the platform, our method produces a constant deviation angle indicating the azimuth angle of the approached position, while Colomb’s method produces a curve with a large change in angle. (TIF) [file pone.0245990.s002.tif]

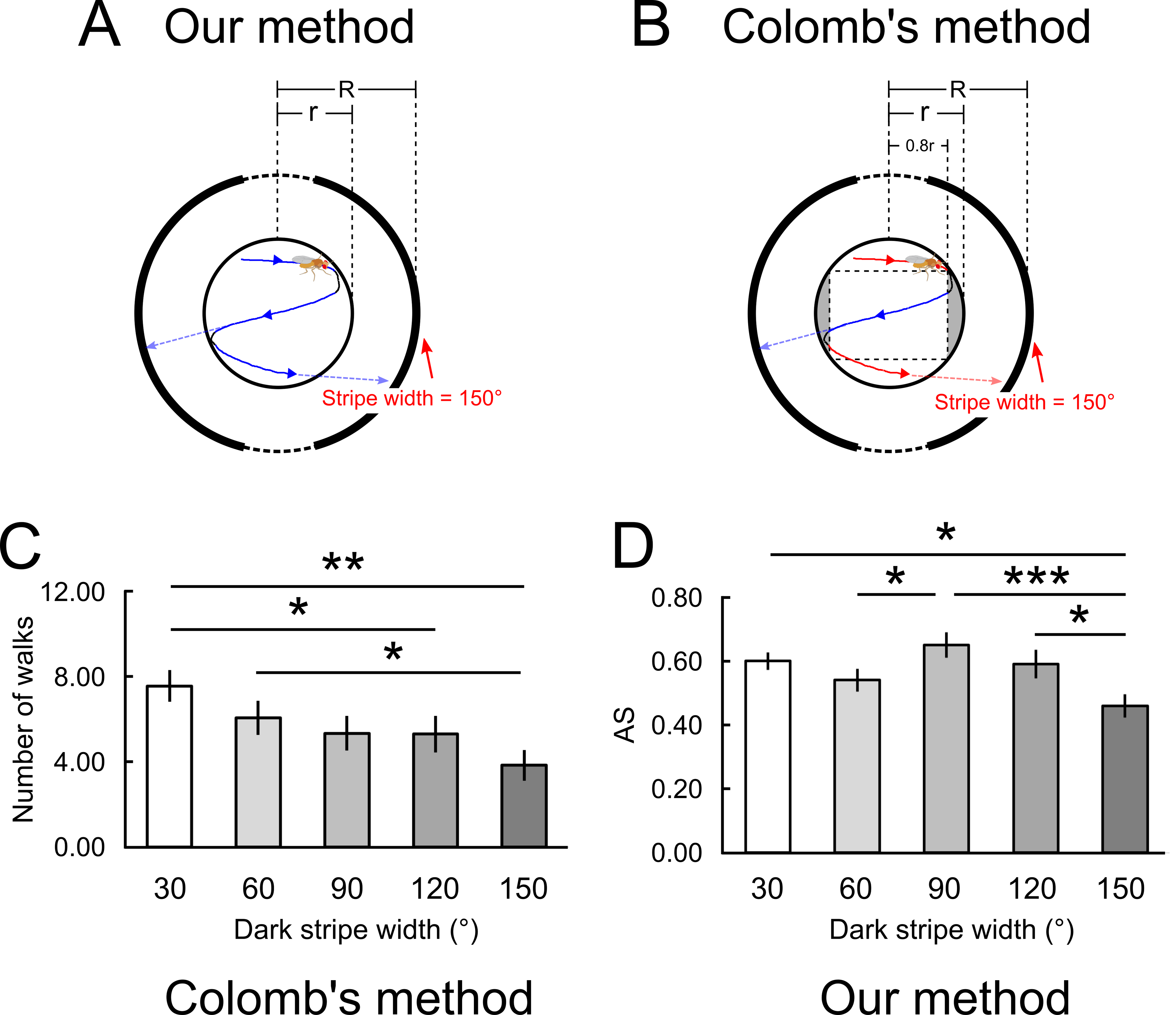

Supplement: S3 Fig — (A) In our method, we first calculate the deviation angles for each video frame and then derive APD and AS from the deviation angles. (B) In Colomb’s method, in addition to calculating the median deviation angle, another metric called “number of walks” was also used. This metric measures the number of walks a fruit fly made between the shaded areas. However, when the stripes become very wide, some walks that still clearly approach the dark stripes may out count in (C) Due to the issue illustrated in (B), the number of walks declines as the width of stripes increases. (D) Our AS still gave rise to large values even for wide stripes. (TIF) [file pone.0245990.s003.tif]

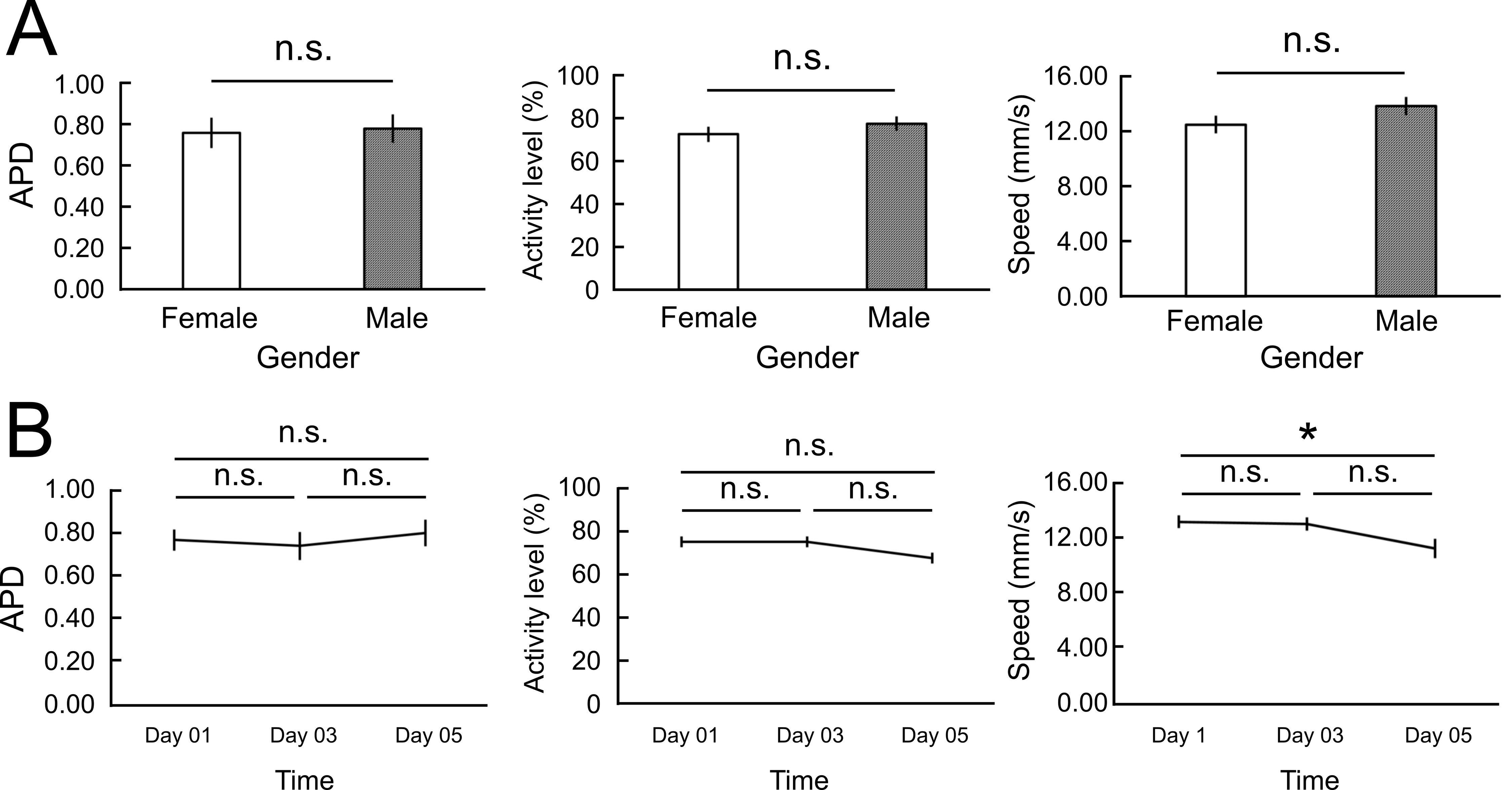

Supplement: S4 Fig — (A) Comparison between female and male for APD, activity level and speed. (B) Comparison between different recovery periods (1, 2 and 3 days). (TIF) [file pone.0245990.s004.tif]

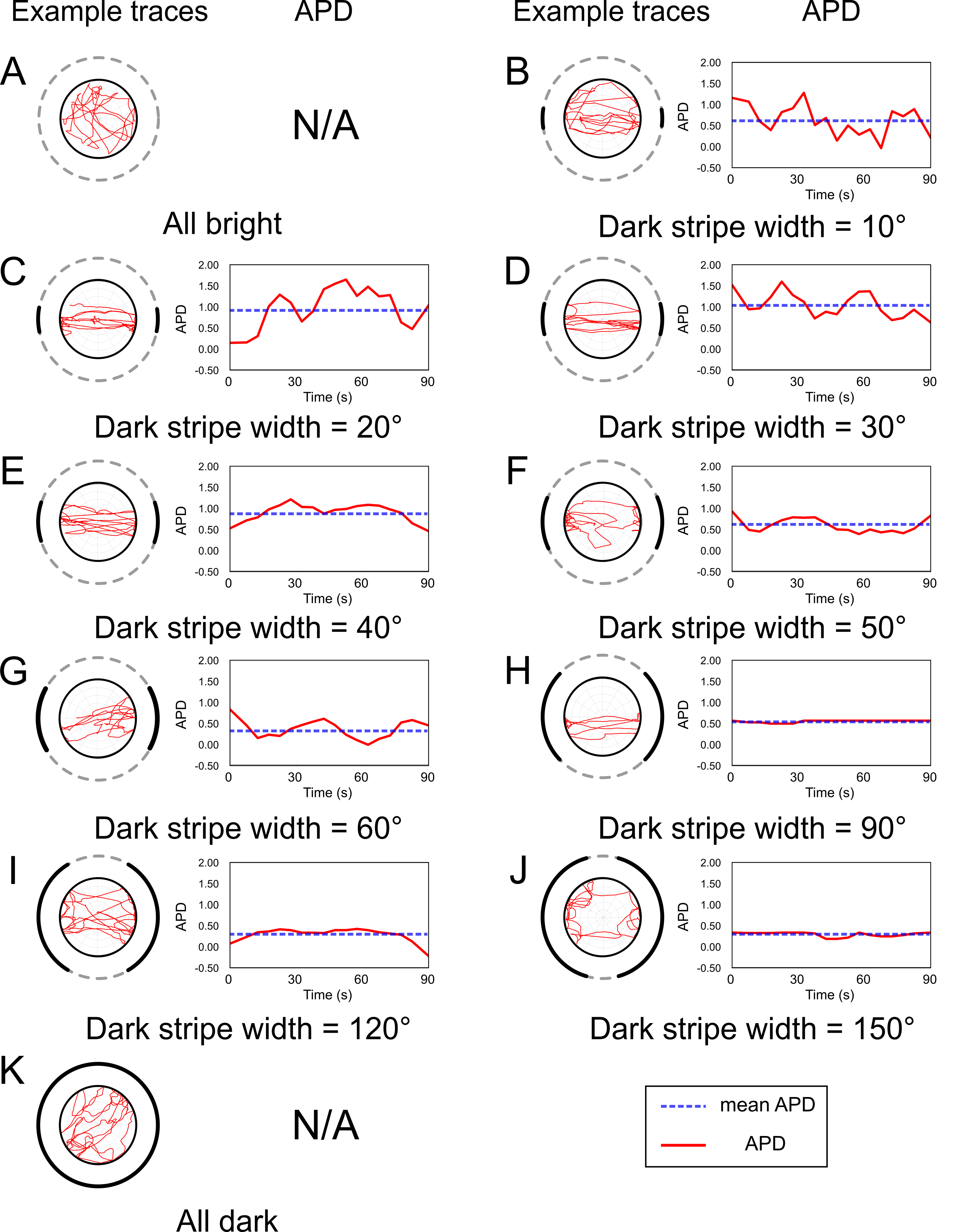

Supplement: S5 Fig — (A) Without any stripe (all-bright condition), the fly did not exhibit any approach behavior. (B)-(J) Example traces and the time-resolved APD for different dark stripe widths. (K) The fly did not exhibit any approach behavior in the all-dark condition. (TIF) [file pone.0245990.s005.tif]

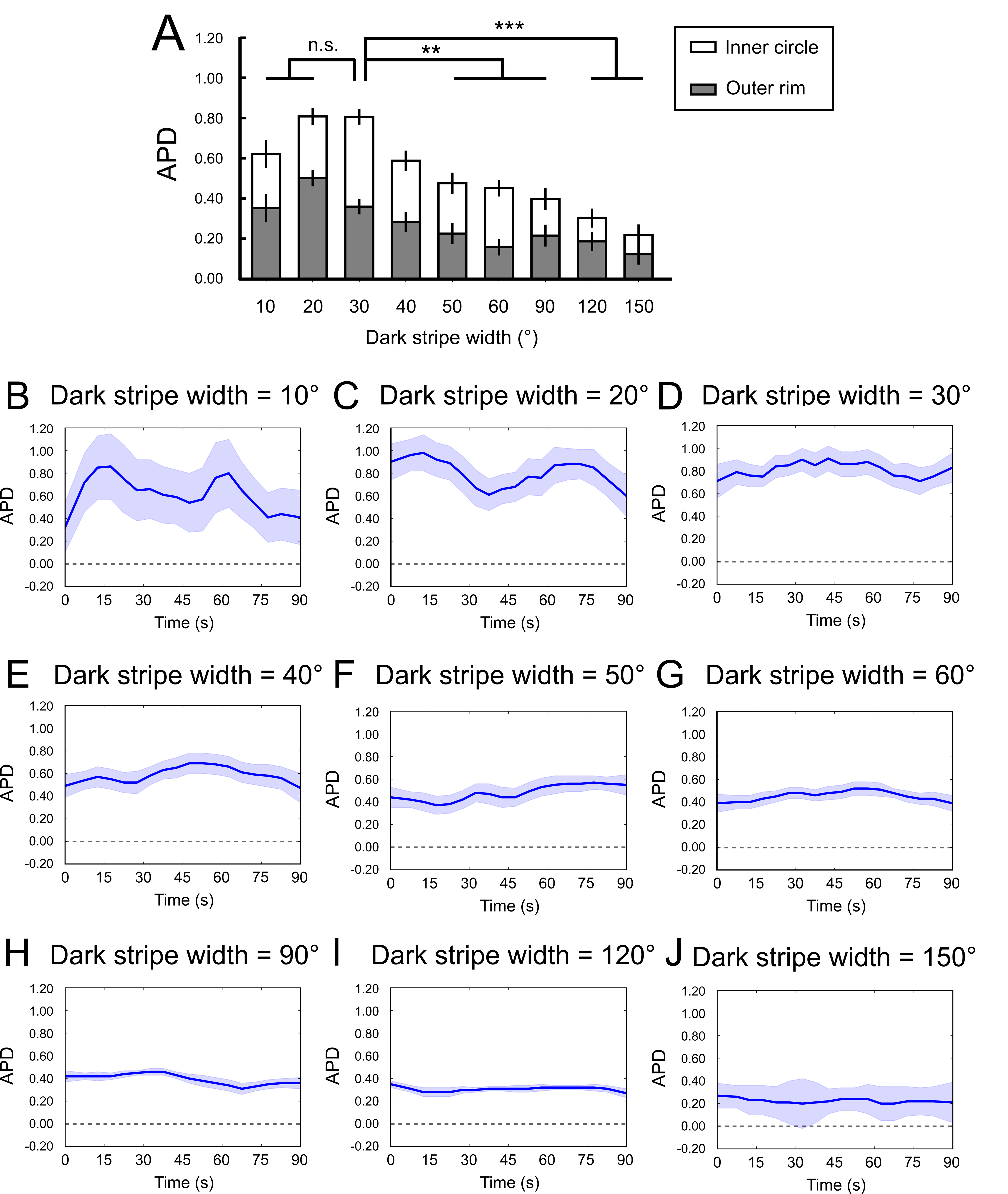

Supplement: S6 Fig — (A) The APD separately calculated for the inner circle and the outer rim. Both regions contribute to the APD significantly. (B)-(J) Time-resolved APD for all tested stripe widths. APD is not defined for the all-bring or all-dark conditions. The shaded area indicates the standard error of the mean. (TIF) [file pone.0245990.s006.tif]

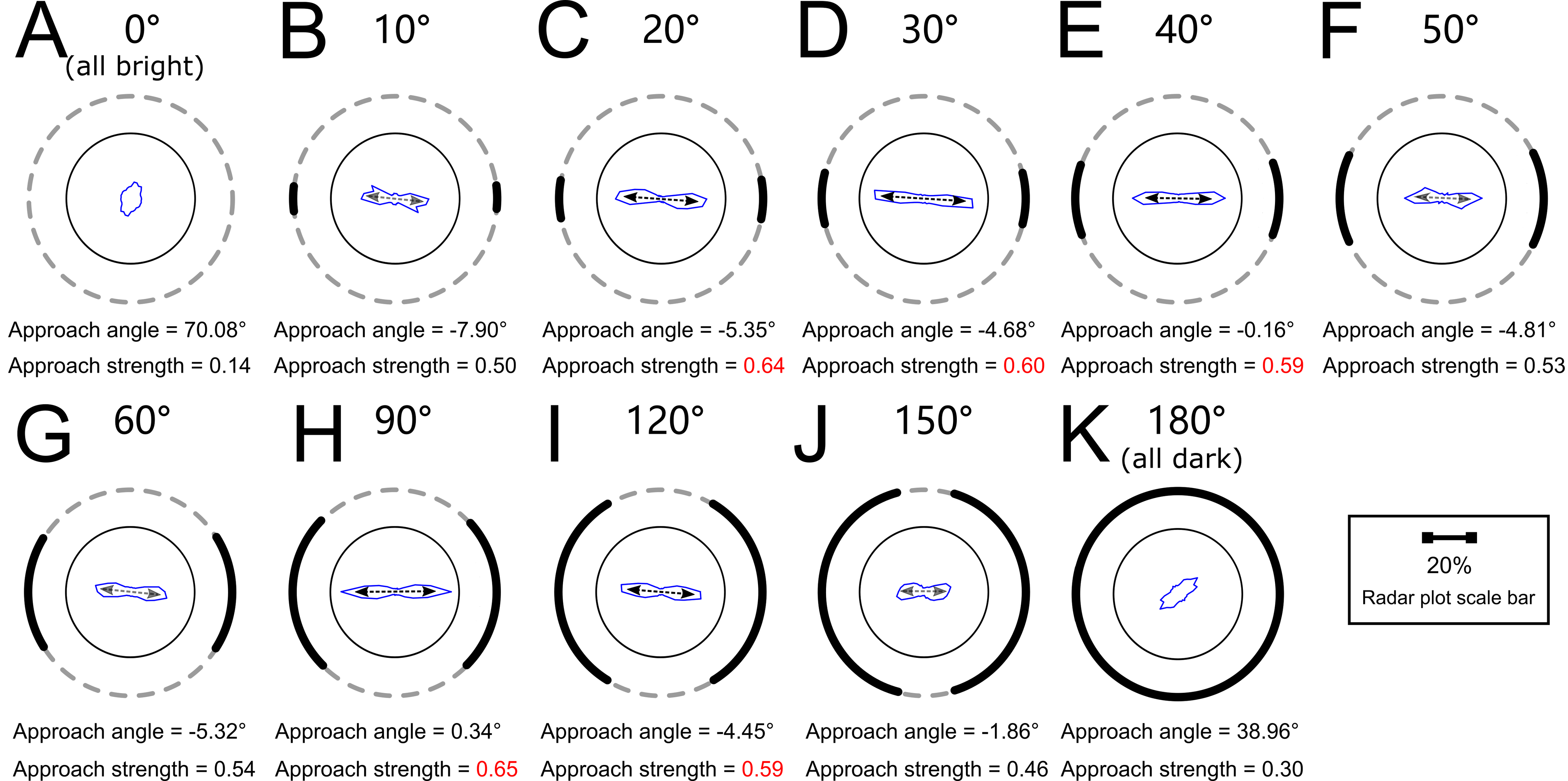

Supplement: S7 Fig — Dark stripes of all sizes elicited significant approach strength with the approach direction toward the center of the dark stripes. (TIF) [file pone.0245990.s007.tif]

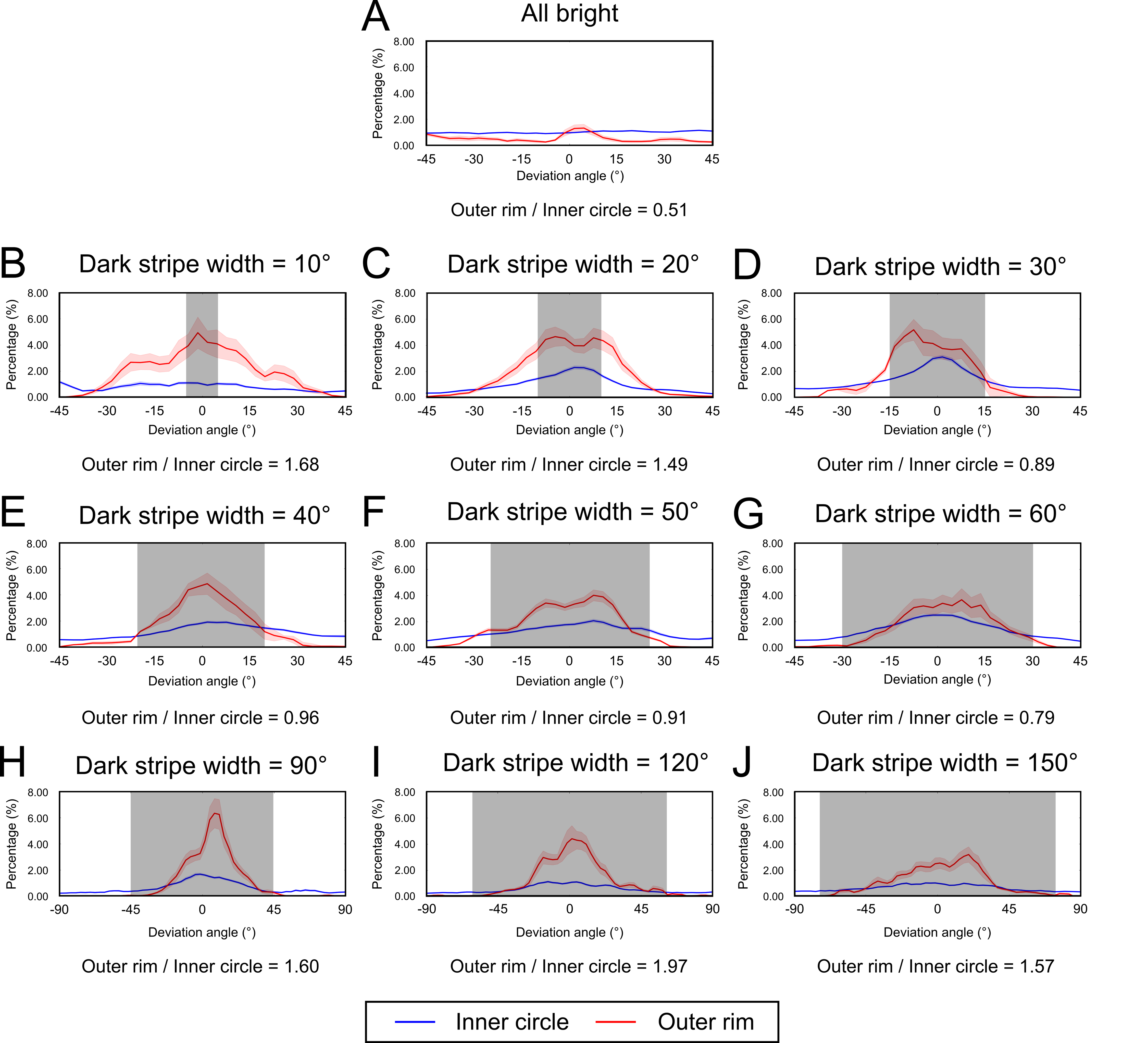

Supplement: S8 Fig — Similar to Fig 5, but both curves in each plot are normalized by the total time spent in the entire arena. Ratio between the time flies spent in the outer rim and inner circle for each condition is labeled under each corresponding plot. (TIF) [file pone.0245990.s008.tif]

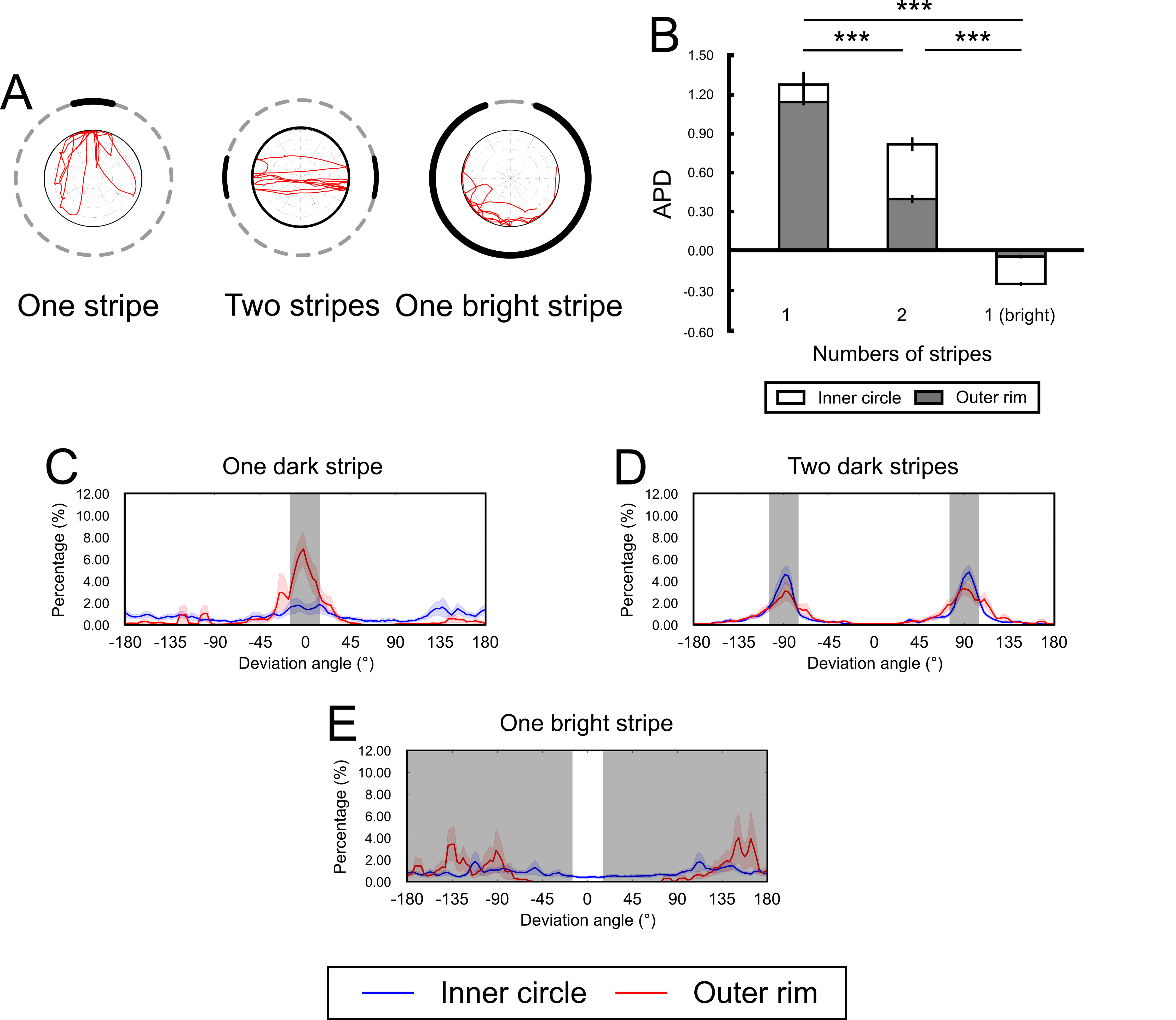

Supplement: S9 Fig — (A) Example movement traces for one dark stripe, two dark stripes (Buridan’s paradigm) and one bright stripe. (B) The population mean APD for all three conditions. (C)-(D) Distributions of deviation angle for all three test conditions. (TIF) [file pone.0245990.s009.tif]
